# NOX2 exacerbates periodontitis via JAK2-STAT3-mediated ferroptosis of gingival epithelial cells

**DOI:** 10.3389/fimmu.2026.1744612

**Published:** 2026-02-26

**Authors:** Yuan Ping, Zimeng Wang, Bo Yang, Mengmeng Li, Lei Li, Xiaonan Zhang, Wei Wang, Yujuan He

**Affiliations:** 1 Department of Laboratory Medicine, Key Laboratory of Diagnostic Medicine (Ministry of Education), Chongqing Medical University, Chongqing, China; 2 The First Affiliated Hospital of Chongqing Medical and pharmaceutical College, Chongqing, China; 3 College of Stomatology, Chongqing Medical University, Chongqing, China

**Keywords:** bioinformatics, ferroptosis, JAK2-STAT3 pathway, NOX2, periodontitis

## Abstract

**Background:**

Ferroptosis is a novel form of regulated cell death driven by lipid peroxidation and oxidative stress, and has been implicated in the pathogenesis of periodontitis. The purpose of this study was to elucidate mechanisms by which NADPH oxidase 2 (NOX2) promotes ferroptosis in gingival epithelial cells and contributes to periodontitis *in vivo*.

**Methods:**

Periodontitis was induced in C57BL/6 mice by silk ligation and an *in vitro* model was established using lipopolysaccharide derived from *Porphyromonas gingivalis* (Pg-LPS) -stimulated CA9–22 gingival epithelial cells. Expression levels of NOX2, GPX4, SLC7A11 and NF-κB and JAK2-STAT3 pathway-related proteins were assessed by Western blotting. Lipid peroxidation was quantified by measuring malondialdehyde (MDA) levels and intracellular reactive oxygen species (ROS) were measured using the fluorescent probe DCFH-DA and detected via microscopy and spectrophotometry. The effects of NOX2 on alveolar bone loss were evaluated by micro-CT analysis and H&E and TRAP staining.

**Results:**

NOX2 expression was significantly elevated in the gingival tissues of periodontitis patients, the mouse model and Pg-LPS-stimulated CA9–22 cells. Mechanistically, we confirmed that Pg-LPS upregulated NOX2 by triggering the TLR4/NF-κB pathway. Gene silencing of NOX2 *in vitro* effectively suppressed ferroptosis as indicated by reduced ROS/MDA levels and restored expression of GPX4 and SLC7A11. Furthermore, H_2_O_2_ added to cell cultures to mimic ROS effects demonstrated that NOX2 mediated ferroptosis via ROS generation and JAK2-STAT3 activation. *In vivo*, pharmacological inhibition of NOX2 attenuated ferroptosis, mitigated alveolar bone loss, and ameliorated periodontal pathology in mice.

**Conclusions:**

NOX2 activation promoted periodontitis by driving ferroptosis via the ROS/JAK2-STAT3 pathway, highlighting its potential as a novel therapeutic target.

## 1 Introduction

Periodontitis is a chronic inflammatory disease triggered by dental plaque that leads to progressive destruction of periodontal supporting tissues, clinically manifested as gingival inflammation, periodontal pocket formation, alveolar bone resorption and tooth loosening and eventual loss (1). The global prevalence of periodontitis has increased significantly and currently affects 1.1 billion people, making it the second most common oral health problem and the sixth most common human disease worldwide (2–4). Periodontitis has also been closely linked to progression of cardiovascular disease, diabetes, rheumatoid arthritis, osteoporosis and cognitive impairment. These diseases seriously affect human oral and systemic health (5, 6). The oral epithelium serves as the first line of defense against periodontal infections and plays a significant role in the pathogenesis of periodontitis (7). Gingival epithelial cells are the first physical and immune barrier of periodontal tissues and damage due to inflammatory stimulation i.e., periodontitis, leads to cell death and destruction of barrier integrity. The loss of epithelial barrier function allows access to bacteria and their metabolites enabling invasion of underlying connective tissue, thereby activating intense innate and adaptive immune responses. These processes aggravate inflammatory destruction of periodontal tissue and alveolar bone resorption that ultimately drives the occurrence and development of periodontitis (8). Therefore, it is necessary to further explore the regulation mechanism of gingival epithelial cell death in periodontitis that which will assist in the identification of intervention targets and to improve the outcome of periodontitis.

Ferroptosis is an iron-dependent form of programmed cell death characterized by phospholipid peroxidation and plasma membrane damage. This process is a key factor in numerous disease states including vascular disorders, cancer, neurodegenerative disease, osteoporosis and diabetes (9–15). Glutathione peroxidase 4 (GPX4) and the cystine-glutamate antiporter system Xc^-^ are the two primary regulators of ferroptosis (16). System Xc^-^ members include the solute carrier family 7 member 11 (SLC7A11) and solute carrier family 3 member 2 (SLC3A2) that inhibit ferroptosis by importing cystine to promote glutathione (GSH) biosynthesis. This enables GPX4 to utilize GSH to reduce elevated ROS and detoxify lipid peroxides (17–19). Recent studies have also demonstrated that ferroptosis plays a significant role in the pathogenesis and progression of periodontitis (20). Clinically, patients with chronic periodontitis demonstrate increased levels of lipid peroxides in saliva and gingival crevicular fluid, concurrent with decreases in GPX activity and the glutathione/oxidized glutathione (GSH/GSSG) ratio in periodontal tissues (21, 22). In experimental periodontitis models, inhibition of ferroptosis in gingival fibroblasts and osteoblasts can reduce Acyl-CoA Synthetase Long Chain Family Member 4 (ACSL4) and lipid peroxide levels, thereby ameliorating periodontal inflammation and tissue destruction (23, 24). Recent studies have reported that *Porphyromonas gingivalis*, a keystone pathogen in periodontitis, weakens the oral epithelial barrier and exacerbates the severity of the disease by inducing ferroptosis through inhibiting the SLC7A11/GSH/GPX4 axis (25). Despite these findings, the role and mechanisms of ferroptosis in periodontitis remain unclear and require further exploration.

Nicotinamide adenine dinucleotide phosphate oxidase 2 (NOX2; *cybb*) is a multi-subunit enzyme complex composed of the transmembrane subunits gp91phox (also known as NOX2) and p22^phox^, cytoplasmic proteins (p47^phox^, p40^phox^ and p67^phox^) and the small GTPase Rac1/2 (26). NOX2 is the terminal component of the respiratory chain that obtains a single electron from cytoplasmic NADPH and transfers it across the plasma membrane to extracellular oxygen to generate reactive oxygen species (ROS) (27). Previous studies have shown that elevated NOX2 expression in periodontitis promotes the production of inflammatory factors and ROS in human gingival fibroblasts (28, 29). Though moderate levels of ROS can resist and eliminate invading pathogenic microorganisms, exceeding the antioxidant threshold leads to increased oxidative load, causing tissue oxidative stress and accelerating periodontal tissue destruction (30, 31). Notably, NOX2-derived ROS not only directly damages biological macromolecules but also binds with polyunsaturated fatty acids (PUFA) to initiate a chain reaction of lipid peroxidation and thereby inducing ferroptosis (32). Recent studies have confirmed that in diabetes, Parkinson’s disease and preeclampsia, NOX2 is aberrantly activated and this exacerbates cell damage through the ferroptosis pathway (33–35). However, the role and molecular mechanisms of NOX2 in periodontitis remain unclear. Consequently, our study aimed to explore whether NOX2 affects periodontitis and damages gingival epithelial cells through ferroptosis. We found that lipopolysaccharide (LPS) derived from *P. gingivalis* (Pg-LPS) promoted NOX2 expression through activation of the TLR4/NF-κB signaling pathway. NOX2 subsequently promoted ferroptosis in gingival epithelial cells via the ROS/JAK2-STAT3 signaling pathway and thereby exacerbated periodontitis progression.

## 2 Materials and methods

### 2.1 Collection of human gingival tissue

Human gingival specimens were procured from the Stomatological Hospital of Chongqing Medical University, comprising healthy controls and patients with periodontitis. None of these selected subjects showed any clinical evidence of recent infection or systemic disease, and none had a history of smoking or maxillofacial surgery, radiotherapy or chemotherapy.

### 2.2 Mice and periodontitis ligation model

C57BL/6 mice (age, 6 weeks old; weight, ≥20 g) were obtained from Chongqing Medical University and housed in a specific pathogen-free environment at constant temperature (22 °C) and humidity (50–60%). The ligature-induced periodontitis model was established by tying 5–0 silk ligatures around the left and right maxillary second molars as previously described (36). The ligatures were kept in position 12 d to promote microbial dental plaque accumulation and inflammation. During this period, the NOX2 inhibitor gp91 ds-tat was injected at 1.5mg/kg into the space between the first molar (M1) and the second molar (M2) using a micro-syringes from the maxillary to buccal side of the oral cavity every 2 d.

### 2.3 Cell culture and treatment

The human gingival cell line CA9–22 was purchased from Otwo Biotech. The experimental cells were initially maintained in DMEM medium (Gibco, Invitrogen, Carlsbad, CA, USA) supplemented with 10% fetal bovine serum (FBS, Biological Industries, Israel), 100 U/ml penicillin and 100 μg/ml streptomycin (Beyotime, Jiangsu, China) at 37°C and 5% CO_2_.

Transient transfection experiments were performed using Lipofectamine 2000 (Invitrogen) and small interfering RNAs (siRNAs) were designed and synthesized by Gene Greate Bio (Wuhan, China) and were applied for 6 hours. The sequences were as follows: 5’-CUACCUAAGAUAGCGGUUGAUdTdT-3’ and 5’-AUCAACCGCUAUCUUAGGUAG dTdT-3’ for NOX2 siRNA, and 5’-UUCUCCGAACGUGUCACGUTT-3’ and 5’- ACGUGACACGUUCGGAGAATT-3’ for negative control siRNA. CA9–22 cells were then stimulated with 20 μg/mL Pg-LPS for 24h in the presence or absence of the following drugs: 400 μM H_2_O_2_, 100 μM PDTC (Pyrrolidinedithiocarbamate ammonium, NF-κB inhibitor), 1 μM TAK-242 (Resatorvid; Toll-like receptor 4 (TLR4) inhibitor), 100 nM Tofacitinib (JAK inhibitor) or 3 μM Fedratinib (JAK2 inhibitor) all obtained from MedChemExpress, Monmouth Junction, NJ, USA. All cell experiments were conducted at least three times independently.

### 2.4 Quantitative real-time PCR

Total RNA was extracted using TRIzol (Invitrogen) according to the manufacturer’s instructions and complementary DNA was synthesized using PrimeScriptRT reagent kit (Takara, Shiga, Japan). Quantitative (q)PCR analysis was performed using the CFX96 real-time PCR detection system (Bio-Rad, Hercules, CA, USA) and an ArtiCan^ATM^ SYBR qPCR Mix (Tsingke Biotech, Beijing, China). Primer sequences for qPCR are listed in **Table 1**.

**Table 1:** Primer sequences used in qRT-PCR experiments.

Gene name	5’-3’	5’-3’
GAPDH (human)	TGCACCACCAACTGCTTAGC	GGCATGGACTGTGGTCATGA
GAPDH (mouse)	TGGAAAGCTGGGCGTGATG	TACTTGGCAGGTTTCTCCAGG
NOX2 (human)	CCTAAGATAGCGGTTGATGG	GACTTGAGAATGGATGCGAA
NOX2 (mouse)	TGTGGTTGGGGCTGAATGTC	CTGAGAAAGGAGAGCAGATTTCG
NOX1	GGAATTAGGCAAAGTGGGTTTT	CAGTGGCCTTGTCAAAGTTTAA
NOX3	CGTGGCGCATTTCTTCAACC	GCTCTCGTTAGGGGTGTTGC
NOX5	CTATTGGACTCACCTGTCCTACC	GGAAAAACAAGATTCCAGGCAC
DUOX1	CCTGGCTCTAGCATGGACAC	CTGCACCTCCCACGAAATG
DUOX2	ACGGTGTGTATCAGGCTCTG	CACGTCGGAAAGAACATGGTAG

The following thermocycling conditions were used for the qPCR: initial denaturation at 95 °C for 5 min; 40 cycles of denaturation at 95 °C for 30 s, annealing at 60 °C for 30 s, and extension at 72 °C for 30 s. Relative expression levels were determined using the 2^-ΔΔCq^ method and normalized to GAPDH.

### 2.5 Western blotting

Total protein was extracted using RIPA lysis buffer and protein concentrations were determined using a BCA Protein Assay Kit (Beyotime, Jiangsu, China). A total of 20 μg protein was separated by SDS-PAGE and electroblotted onto PVDF membranes. The membranes were incubated with the following primary antibodies overnight at 4 °C: NOX2 (1:2000, Huabio, China), GPX4 (1:10000, Huabio), SLC7A11 (1:1000, Huabio), JAK2 (1:1000, Huabio), STAT3 (1:1000, Huabio), TLR4 (1:1000, Zenbio, China), phospho-JAK2 (1:1000, Zenbio), phospho-STAT3 (1:1000, Zenbio), NF-κB p65 (1:1000, Cell Signaling Technology, Beverley, MA, USA), phospho-NF-κB p65 (1:1000, Cell Signaling), GAPDH (1:10000, Proteintech Biotechnology, China) and α-tubulin (1:10000, Proteintech). Following the primary antibody incubation, membranes were incubated with HRP-conjugated goat anti-rabbit IgG (H + L) (1:10000, Huabio) or HRP-conjugated goat anti-mouse IgG (H + L) (1:10000, Huabio) at room temperature for 1 h. The results were visualized with a chemiluminescence imaging system and further analyzed by ImageJ software Version 2.9.0 (NIH, Bethesda, MD, USA; https://imagej.net/ij/).

### 2.6 ROS measurement

Intracellular ROS levels were determined using 2’,7’-dichlorofluorescin diacetate (DCFH-DA, Beyotime). DCFH-DA was diluted with serum-free DMEM to a working solution (1:1000) and cells were incubated with this solution at 37°C for 20 min. After 3 washes with culture medium, cells were observed and photographed using a fluorescence microscope (Nikon, Melville, NY, USA). Finally, the collected cell suspensions were analyze using a fluorescence spectrophotometer (UV-1600, Shanghai Mapada Instruments Co., Ltd.).

### 2.7 MDA assay

A Lipid Peroxidation Assay Kit (Beyotime) was used to evaluate the relative concentration of malondialdehyde (MDA) in tissue and cell lysates following the manufacturer’s instructions. MDA was measured at 532 nm via the thiobarbituric acid (TBA) method. The concentrations were calculated based on the standard curve and normalized to corresponding protein concentration.

### 2.8 Cell viability assay

Cell viability was assessed using the Cell Counting Kit-8 (CCK-8, Beyotime, China) according to the manufacturer’s instructions. In brief, CA9–22 cells were seeded into 96-well plates for drug treatment experiments. Following incubation, 10 μL CCK8 solution was added and allowed to stand for 1 h at 37°C in the dark. The absorbance at 450 nm was measured using a microplate reader, and the optical density values of different groups were used to determine cell viability.

### 2.9 Immunohistochemistry staining

Paraffin-embedded sections were deparaffinized, subjected to antigen retrieval via microwave heating, and treated with 3% H_2_O_2_ to block endogenous peroxidase activity. After blocking with 3% bovine serum albumin (BSA), sections were incubated overnight at 4°C with anti-NOX2 primary antibody (1:200, Servicebio, China), followed by incubation with the secondary antibody. Sections were then developed with DAB and counterstained with hematoxylin.

### 2.10 Immunofluorescence staining

Cellular immunofluorescence experiments utilized CA9–22 cells fixed with 4% paraformaldehyde (PFA) and washed with PBS for three times. The cells were then permeabilized and blocked using 0.5% Triton X-100 and 5% BSA, respectively and then incubated with anti-NOX2 primary antibody (1:500, Proteintech) at 4 °C overnight and with fluorescence dye-conjugated secondary antibodies at 37 °C 1 h. Nuclei were stained with DAPI for 15 min. Images were captured under a fluorescent microscope (Nikon).

Immuno-histo-fluorescence utilized mouse maxillae sections that were incubated with anti-GPX4 (1:200, Huabio) and anti-SLC7A11(1:200, Huabio) primary antibodies overnight at 4°C, followed by incubation with the secondary antibodies. Nuclei were counterstained with DAPI.

### 2.11 Histological analysis

Hematoxylin and eosin (H&E) staining utilized paraffin-embedded sections sequentially stained with H&E to visualize nuclei and cytoplasm, respectively. For TRAP staining, the sections were stained using the Trap stain kit (Solarbio, Beijing, China) in accordance with the manufacturer’s instructions. Images were obtained using a microscope slide scanner. Osteoclasts with Trap positivity in the alveolar bone around the maxillary second molar were analyzed using ImageJ software.

### 2.12 Micro-CT analysis

The entire maxillary molars of C57BL/6 mice were removed and analyzed using a micro-CT system (Nemo, Pingseng Scientific, China). The rebuilt images of bone surfaces were used to perform three-dimensional histomorphometric analyses using the same density.

### 2.13 Bioinformatic analysis

We initially consulted a microarray data set that encompassed 241 patients with periodontitis and 69 controls for this study that were obtained from the GEO database (GSE16134) (https://www.ncbi.nlm.nih.gov/geo/) (37, 38). R software was used for data processing, analysis, and figure generation. Using thresholds of |log_2_FC| > 0.5 and adj. p < 0.05, differentially expressed genes (DEGs) were visualized as a volcano plot using “ggplot2”, while a heatmap of the DEGs was created with the “pheatmap” package (39, 40). “WGCNA” package was utilized to generate a heatmap depicting the correlations between gene modules and clinical traits (41). A proteomic dataset of gingival crevicular fluid from patients with periodontitis was obtained from the PRIDE database under the accession number PXD046328 (42, 43). Ferroptosis-related genes were retrieved from the FerrDb database (44). The STRING database (http://string-db.org, version 11.5) online tool was used to predict and visualize PPI network models based on the seven screened ferroptosis-related genes found to be active in patients with periodontitis (45). Functional enrichment analysis was performed and visualized using the “GOplot” package in R. The “ggpubr” R package was employed to produce violin plots illustrating gene expression levels in both control and periodontitis samples. Receiver operating characteristic (ROC) curves were generated using the “pROC” package in R.

### 2.14 Statistical analysis

All results are presented as mean ± standard error of measurement (SEM). Data were analyzed with Prism (Version 8.0, GraphPad, Boston, MA, USA). The t-test and one-way ANOVA were used for statistical analysis of data between two or more groups. p < 0.05 was considered as being statistically significant (ns = not significant, * p < 0.05, ** p < 0.01, *** p < 0.001, **** p < 0.0001).

## 3 Results

### 3.1 Identification of *cybb* as a key ferroptosis-related gene in periodontitis

**Figure 1:**
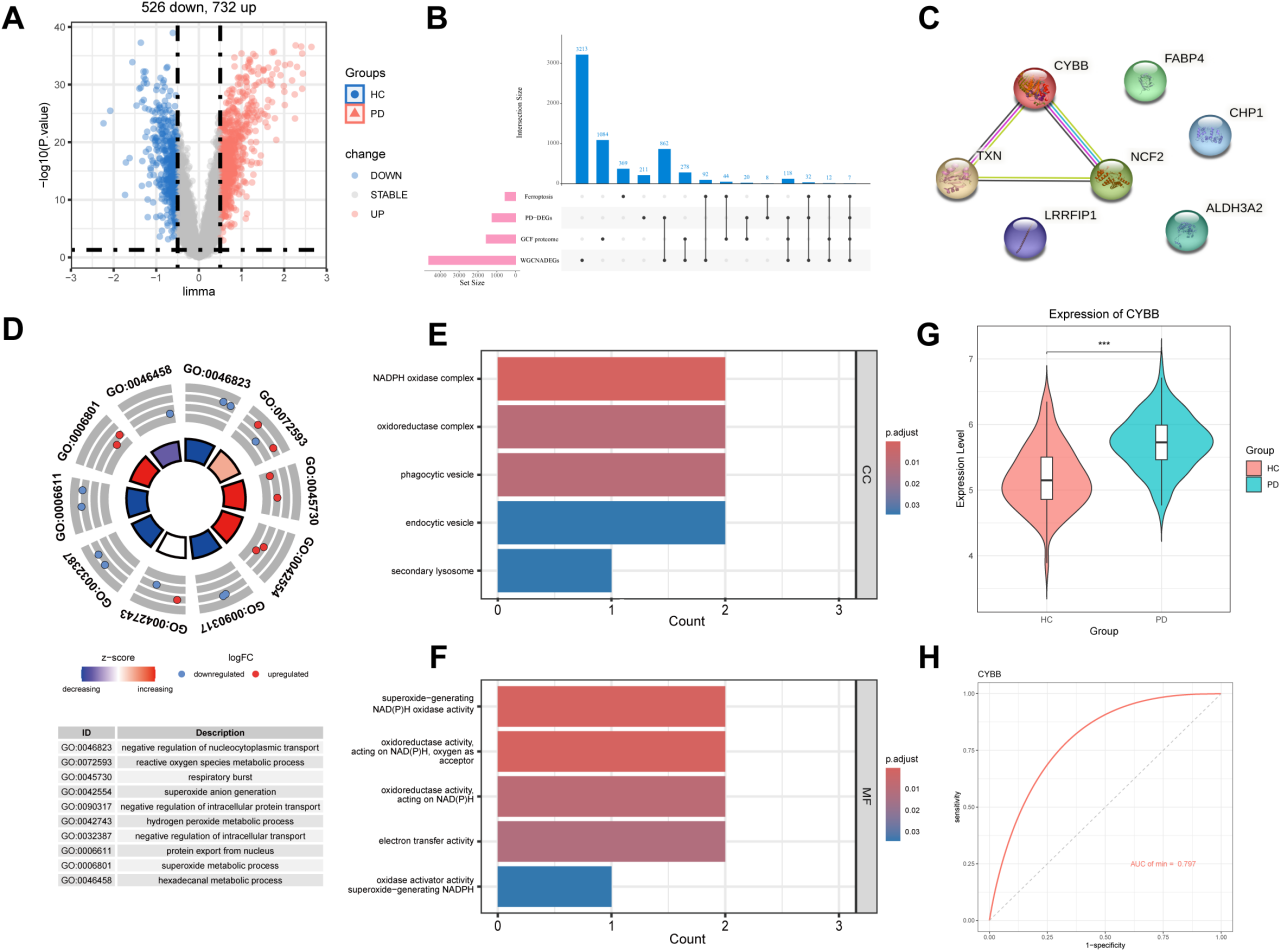
Identification of *cybb* as a key ferroptosis-related gene in periodontitis. **(A)** Volcano plot of differential gene expression in the GSE16134 dataset. **(B)** The upset plot of ferroptosis-related genes in periodontitis. **(C)** Protein correlation analysis of 7 hub genes using String database. **(D–F)** GO functional enrichment analysis (including BP, MF and CC). **(G)** Relative gene expression level of NOX2 in the gingival tissues of patients with periodontitis compared with healthy controls (using dataset GSE16134; control, n = 69; periodontitis, n = 241). **(H)** Receiver operating characteristic curve analysis of NOX2 in GSE16134.

We initially examined a large GEO microarray dataset (GSE16134) that encompassed 241 patients with periodontitis and 69 healthy controls and identified differentially expressed genes (DEGs) between the two groups. We found 526 downregulated and 732 upregulated DEGs (**Figure 1A**). We then applied a WGCNA analysis to correlate DEGs with clinical traits and identifiedfour key modules (blue, light yellow, magenta, dark red) with correlation coefficients ≥0.75 (**Supplementary Figures S1A–E**). We additionally analyzed a proteomic dataset (PXD046328) that included 1536 gingival crevicular fluid proteins from periodontitis patients (42, 43) as well as ferroptosis-related genes that were retrieved from the FerrDb database. These data were intersected and this resulted in the identification of 7 genes that overlapped between the test categories (**Figure 1B**). Simultaneously, we constructed a protein-protein interaction network analysis on these genes using STRING database (**Figure 1C**). Subsequent GO enrichment analysis of the 7 genes revealed significant categories related to the following: biological processes (negative regulation of nucleocytoplasmic transport, reactive oxygen species metabolic process and respiratory burst); cellular components (NADPH oxidase complex) and molecular function (superoxide-generating NAD(P)H oxidase activity) (**Figures 1D–F**). These findings indicated a crucial role of oxidative stress and NADPH oxidase-mediated mechanisms in periodontitis. We therefore focused on *cybb* (encoding NOX2) that was upregulated in periodontitis (**Figure 1G**) and that demonstrated a potential for use as a diagnostic tool (AUC = 0.797; **Figure 1H**).

### 3.2 NOX2 expression was upregulated in periodontitis

**Figure 2:**
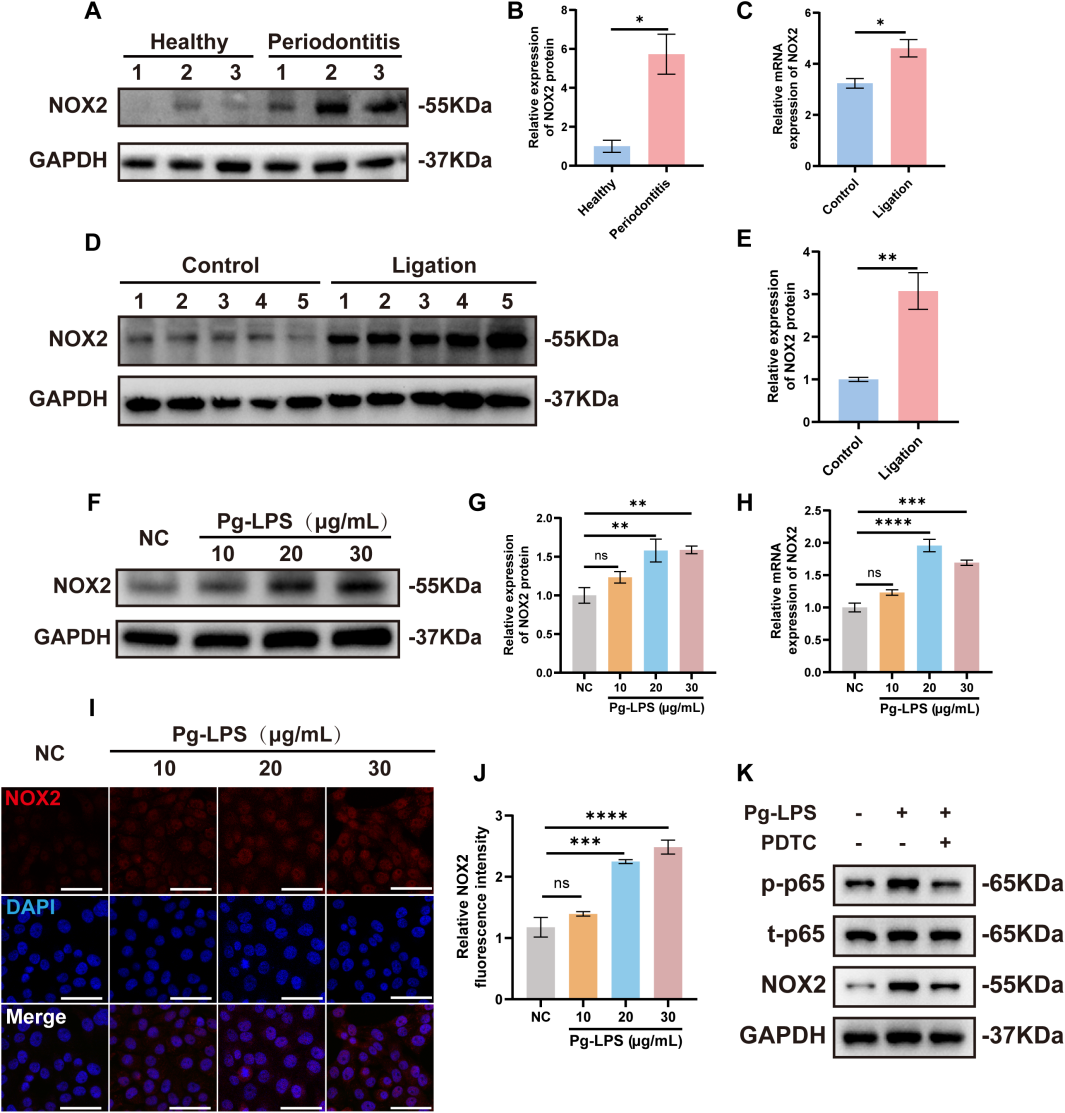
NOX2 expression is upregulated in periodontitis. **(A)** Western blot analysis of NOX2 expression in gingival tissues from patients with periodontitis and healthy controls (n=3). **(B)** Quantitative analysis of WB results in **(A)**. **(C)** RNA and proteins were extracted from mouse gingival tissues for the Control group and the Ligation group (n=5). The mRNA **(C)** and protein **(D, E)** levels of NOX2 were detected by RT-qPCR and Western blot, respectively. **(F, G)** Protein and **(H)** mRNA levels of NOX2 in CA9–22 cells stimulated by Pg-LPS (10, 20 30 μg/mL) for 24 h (n=3). **(I)** Representative immunofluorescence images of CA9–22 cells stimulated by Pg-LPS (NOX2 in red, nuclear in blue, Scale bars: 50 μm). **(J)** Quantitative analysis of NOX2 fluorescence intensity (n=3). **(K)** CA9–22 cells were pretreated with PDTC (100 μM) for 1 h before the treatment of 20 μg/mL Pg-LPS for 24 h (n=3); NOX2 and phosphorylation level of p65 was measured by Western blot. Data are presented as mean ± SEM. Statistical analysis was performed using the unpaired two-tailed Student’s t-test for comparisons between two groups, and one-way ANOVA followed by Tukey’s *post hoc* test for comparisons among multiple groups. *p < 0.05, **p < 0.01, ***p < 0.001 and ****p < 0.0001.

We further investigated whether NOX2 played a role in the pathology of periodontitis and examined its expression across clinical and experimental models. In human gingival tissues, NOX2 protein levels were significantly elevated in patients with periodontitis compared to healthy controls. (**Figures 2A, B**). In a mouse model of periodontitis, both NOX2 mRNA and protein expression were markedly increased (**Figures 2C–E**). In gingival epithelial cells stimulated with Pg-LPS, NOX2 expression was upregulated at both the transcriptional and translational levels in a concentration-dependent manner (**Figures 2F–J**). These results indicated that NOX2 expression was upregulated in periodontitis. Our resultsdemonstrated that Pg-LPS stimulation elevated TLR4 and p-p65 levels in a concentration- andtime-dependent manner similar to two other studies (46, 47) (**Supplementary Figures S2A, B**). To experimentally confirm a linkage between NOX2 expression and periodontitis, we treatedCA9–22 cells with Pg-LPS and examined NOX2 protein expression via Western blotting. Pg-LPSaddition induced NOX2 expression while pretreatment with the TLR4 inhibitor TAK-242 or the NF-κB inhibitor PDTC both significantly suppressed this upregulation (**Supplementary Figures S2C**, **2K**). Together, these findings demonstrated that Pg-LPS upregulated NOX2 expression via the TLR4/NF-κB signaling pathway.

### 3.3 NOX2 silencing suppressed Pg-LPS-induced ferroptosis in CA9–22 cells

**Figure 3:**
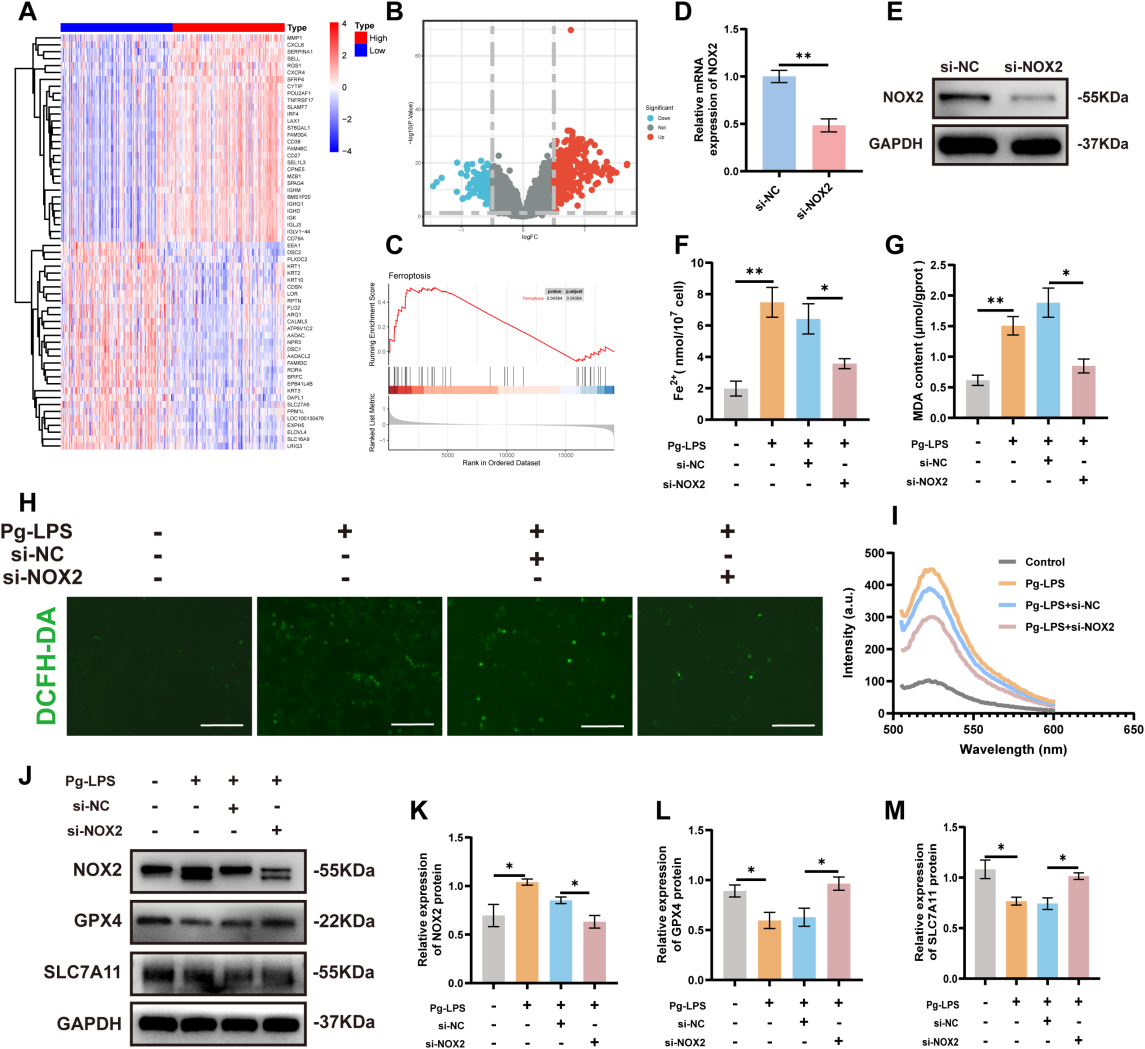
NOX2 silencing suppresses Pg-LPS-induced ferroptosis in CA9–22 cells. **(A)** Heat map representing the 30 most highly-expressed up-regulated and down-regulated DEGs related to NOX2 expression in GSE16134. **(B)** Volcano plot of DEGs between high and low NOX2 expressing periodontitis samples (GSE16134). **(C)** GSEA plot showing enrichment of the “*Ferroptosis*” in the NOX2 high expression group of patients with periodontitis in GSE16134. **(D)** The efficiency of si-NOX2 in CA9–22 cells verified using RT-qPCR measurements of mRNA levels (n=3). **(E)** NOX2 protein levels in CA9–22 cells transfected with si-NOX2 was measured by Western blotting. **(F)** The Fe^2+^ levels in CA9–22 cells (n=3). **(G)** Relative levels of MDA in CA9–22 cells (n=3). **(H)** Representative images of ROS evaluated by DCFH-DA staining (Scale bars: 500 μm). **(I)** Levels of ROS measured by fluorescence spectrophotometry. **(J–M)** GPX4 and SLC7A11 protein levels in CA9–22 cells after NOX2 knockdown. Mean ± SEM (n=3). Statistical analysis was performed using one-way ANOVA with Tukey’s *post hoc* test. *p < 0.05, **p < 0.01.

Human gingival tissue samples from periodontitis patients (GSE16134) were divided based on median NOX2 expression to enable gene expression profile analysis (**Figures 3A, B**). GSEA identified significant enrichment of “*Ferroptosis*” in tissues with high NOX2 expression indicating a correlation between NOX2 and ferroptosis in periodontitis (**Figure 3C**). To explore the biological role of NOX2, we used siRNA gene silencing to target the NOX2 gene in CA9–22 cells. We found significant suppression of NOX2 expression following NOX2 siRNA transfection (**Figures 3D, E**). To assess the intracellular labile iron levels, we found that intracellular Fe^2+^ increased significantly following Pg-LPS stimulation and knockdown of NOX2 reduced Fe^2+^ levels compared to the si-NC group (**Figure 3F**). Moreover, Pg-LPS induction significantly increased MDA levels compared with controls and the effect was reversed upon transfection with si-NOX2 (**Figure 3G**). Pg-LPS also significantly raised cellular ROS levels and these could be reduced by downregulating NOX2 with siRNA (**Figures 3H, I**). Additionally, data from Western blotting demonstrated that GPX4 and SLC7A11 expression levels in Pg-LPS induced CA9–22 cells were conspicuously reduced vs. controls and these effects were all reversed following transfection with si-NOX2 (**Figures 3J–M**). These findings demonstrated that NOX2 silencing suppressed Pg-LPS-induced ferroptosis in CA9–22 cells.

### 3.4 NOX2-mediated ROS regulated ferroptosis via the JAK2-STAT3 signaling pathway

**Figure 4:**
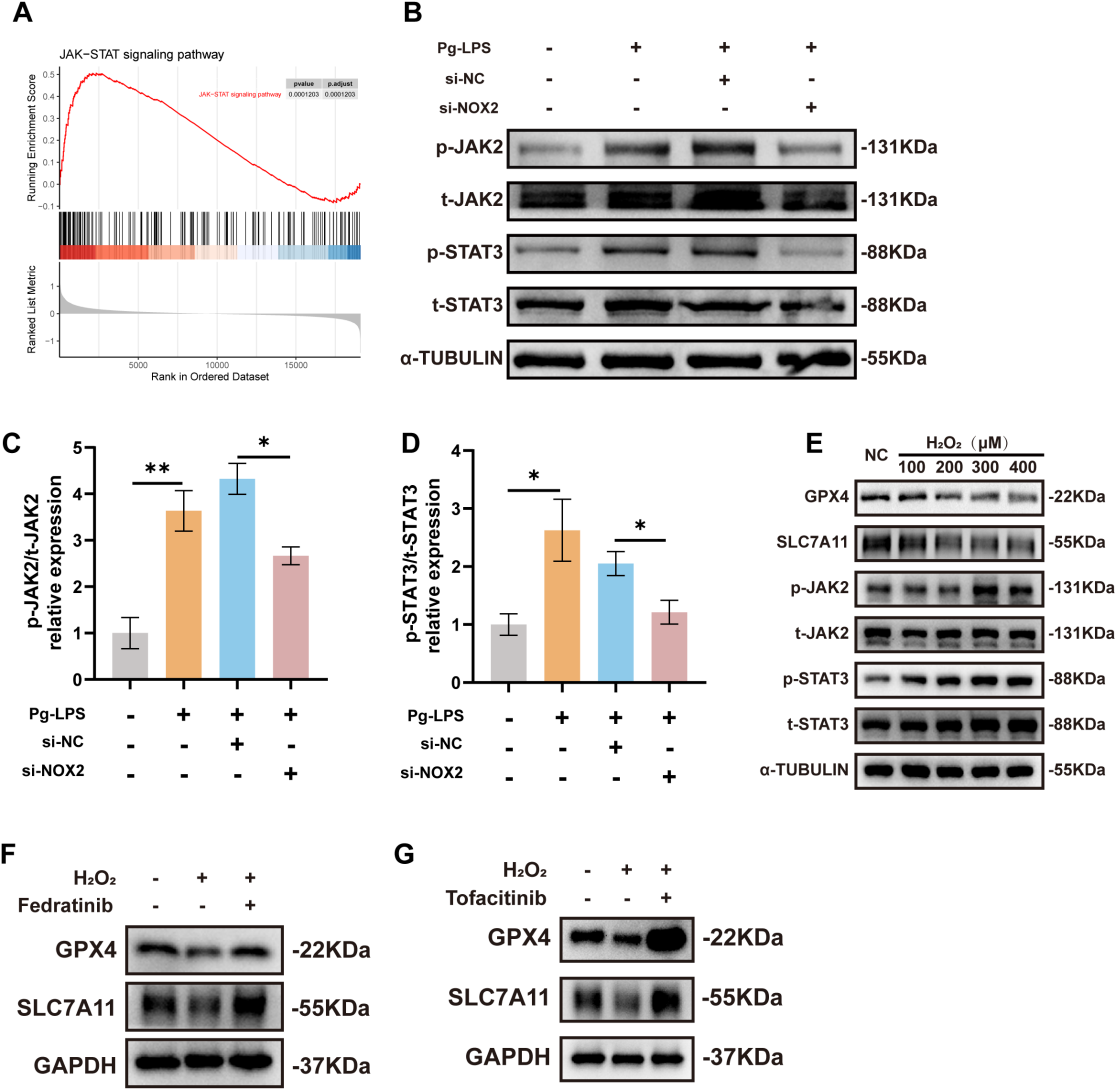
NOX2-mediated ROS regulated ferroptosis via the JAK2-STAT3 signaling pathway. **(A)** GSEA plot depicting enrichment of the “*JAK-STAT signaling pathway*” in the NOX2 high expression group of patients with periodontitis in GSE16134. **(B–D)** Phosphorylation levels of JAK2 and STAT3 in CA9–22 cells following NOX2 gene silencing (n=3). **(E)** Western blot analysis of GPX4, SLC7A11, p-JAK2, and p-STAT3 protein levels in CA9–22 cells treated with H_2_O_2_ (100, 200, 300 and 400 μM) for 6 h (n=3). **(F, G)** CA9–22 cells were respectively pretreated with Fedratinib (3 μM) and Tofacitinib (100 nM) for 1 h prior to the treatment with 400 μM H_2_O_2_ for 6 h; levels of GPX4 and SLC7A11 were measured by Western blotting (n=3). Data are presented as mean ± SEM (n=3). Statistical analysis was performed using one-way ANOVA with Tukey’s *post hoc* test. *p < 0.05, **p < 0.01.

GSEA also identified significant enrichment of the “*JAK-STAT signaling pathway*” in tissues with high NOX2 expression (**Figure 4A**) and indicated a correlation between NOX2 and JAK-STAT signaling in periodontitis.Considering the active involvement of the JAK2-STAT3 signaling pathway in ferroptosis andperiodontitis pathogenesis (48, 49), we further investigated whether NOX2 silencing provided a protective mechanism. We therefore examined JAK2 and STAT3 and phospho-JAK1 levels in Pg-LPS stimulated CA9–22 cells. JAK1 phosphorylation was not significantly activated confirming that this JAK isoform was not involved and further supporting the specificity of Pg-LPS in activating JAK2 (**Supplementary Figure S3A**). In contrast, NOX2 silencing significantly reduced Pg-LPS-induced increases in the levels of phosphorylated JAK2 and STAT3 (**Figures 4B–D**). Additionally, since NOX isoforms play central roles in catalytic ROS generation, we soughtto determine whether NOX2-mediated ROS was responsible for these alterations in JAK2-STAT3activation. We first examined the expression levels of other NOX subtypes (excluding NOX2) in response to Pg-LPS stimulation and we found no significant changes in any of these genes indicating that the ROS induced by Pg-LPS primarily originates from NOX2 (**Supplementary Figure S4**). Furthermore, we treated CA9–22 cells with exogenous H_2_O_2_ to mimic ROS effects *in vitro*. As H_2_O_2_ levels increased, the levels of the ferroptosis-related proteins GPX4 and SLC7A11 declined, while phosphorylation levels of JAK2 and STAT3 were elevated (**Figure 4E**). This implicated ROS-induced activation of the JAK2–STAT3 pathway. To verify therole of JAK2-STAT3 in ferroptosis, the JAK2-specific inhibitor Fedratinib and JAK inhibitorTofacitinib were employed (**Supplementary Figure S5A**). Fedratinib effectively inhibited p-JAK2 and p-STAT3 expression (**Supplementary Figure S5B**). In H_2_O_2_-treated CA9–22 cells, GPX4 and SLC7A11 expression levels were markedly decreased. These effects were reversed by pretreatment with either Fedratinib or Tofacitinib (**Figures 4F, G**). These findings demonstrated that NOX2 mediates ferroptosis *in vitro* by producing ROS and subsequently modulating the JAK2–STAT3 pathway.

### 3.5 Inhibition of NOX2 ameliorated periodontitis by suppressing ferroptosis in mice

**Figure 5:**
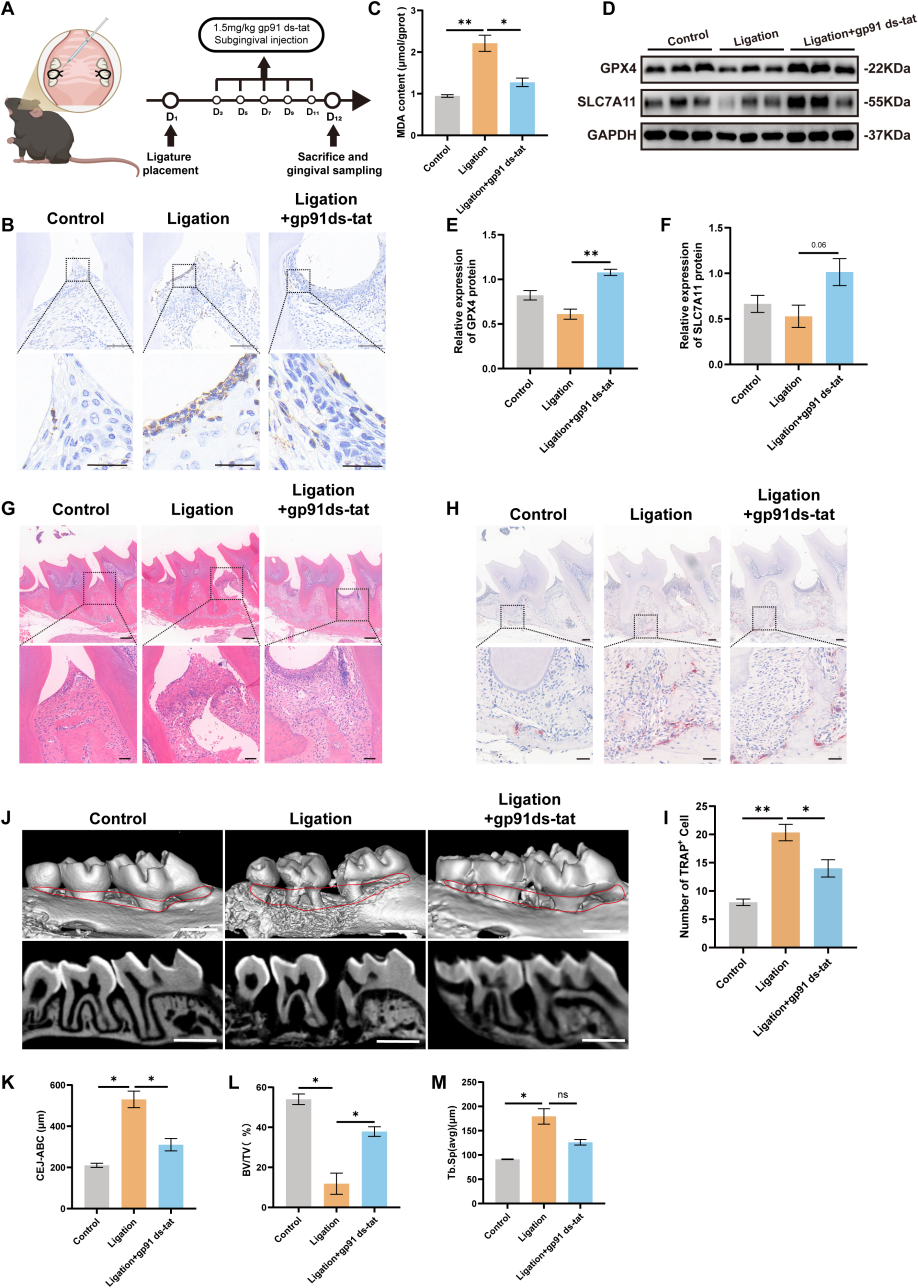
Inhibition of NOX2 ameliorated periodontitis by suppressing ferroptosis in mice. **(A)** Flowchart of the experimental protocol. **(B)** Representative images of immunohistochemical staining of NOX2 from mice. Scale bars, 100 μm (upper); 15 μm (lower). **(C)** Relative MDA levels in gingival tissues (n=3). **(D–F)** GPX4 and SLC7A11 protein levels in mice for the indicated groups (n=3). **(G)** Representative H&E staining images of histological sections of the interproximal area between the 1st and 2nd molars for each experimental group Scale bars, 200 μm (upper); 20 μm (lower). **(H)** Representative TRAP-stained sections of gingival tissues Scale bars, 100 μm (upper); 20 μm (lower). **(I)** Number of TRAP-positive cells at the ligature site (n=3). **(J)** Representative micro-CT reconstruction images of the maxillary molars. The red line range corresponds to the distances of CEJ-ABC (Scale bars: 1 mm) (n=3). **(K–M)** Measurements of CEJ -ABC, BV/TV and Tb.Sp for each group. BV/TV: bone tissue volume/tissue volume (%); CEJ-ABC: cementoenamel junction and alveolar bone crest; Tb.Sp: trabecular separation. Data are presented as mean ± SEM (n=3). Statistical analysis was performed using one-way ANOVA with Tukey’s *post hoc* test. *p < 0.05, **p < 0.01.

To elucidate a role for NOX2 in periodontitis *in vivo*, we administered the NOX2 inhibitor gp91 ds-tat in the experimentally-induced mouse model of periodontitis (**Figure 5A**). Immunohistochemistry staining revealed a significant increase in NOX2 in the ligature group vs. controls while gp91 ds-tat treatment reduced NOX2 expression (**Figure 5B**). Lipid peroxidation assays also indicated that MDA levels were elevated in periodontitis mice and this could be attenuated by NOX2 inhibition (**Figure 5C**). The ligature-induced mice also exhibited reduced GPX4 and SLC7A11 protein levels vs. controls and gp91 ds-tat treatment reversed these effects (**Figures 5D–F**). These findings were further corroborated using immunofluorescence staining of the cells(**Supplementary Figures S6A–D**).

Together, these findings indicated that NOX2 inhibition reduced ferroptosis in mice with periodontitis. We next investigated whether a down-regulation of NOX2 could mitigate periodontitis. The gp91 ds-tat treatment group had a thicker periodontal epithelial fibrous layer, better alveolar bone height and morphology and fewer infiltrating inflammatory cells than did the ligation group (**Figure 5G**). Since osteoclasts are the primary participant in bone resorption, we examined whether there was an imbalance in the osteoclast population. We found fewer osteoclasts in periodontal tissue in the gp91ds-tat group than in the ligation group using TRAP staining (**Figures 5H, I**). A micro-CT analysis also indicated obvious alveolar bone resorption in the ligation group compared with controls and interestingly, gp91 ds-tat treatment reversed this condition (**Figure 5J**). Furthermore, ligation induced significant increases in both cementoenamel junction-alveolar bone crest (CEJ-ABC) distance and trabecular spacing (Tb.Sp) while the bone volume fraction (BV/TV) was decreased. These pathological changes were effectively normalized by gp91 ds-tat administration where CEJ-ABC measurements and Tb.Sp were decreased while BV/TV levels were increased (**Figures 5K–M**). Taken together, these findings indicated that NOX2 inhibition may confer a protective effect by suppressing ferroptosis in the ligature-induced periodontitis model.

## 4 Discussion

**Figure 6:**
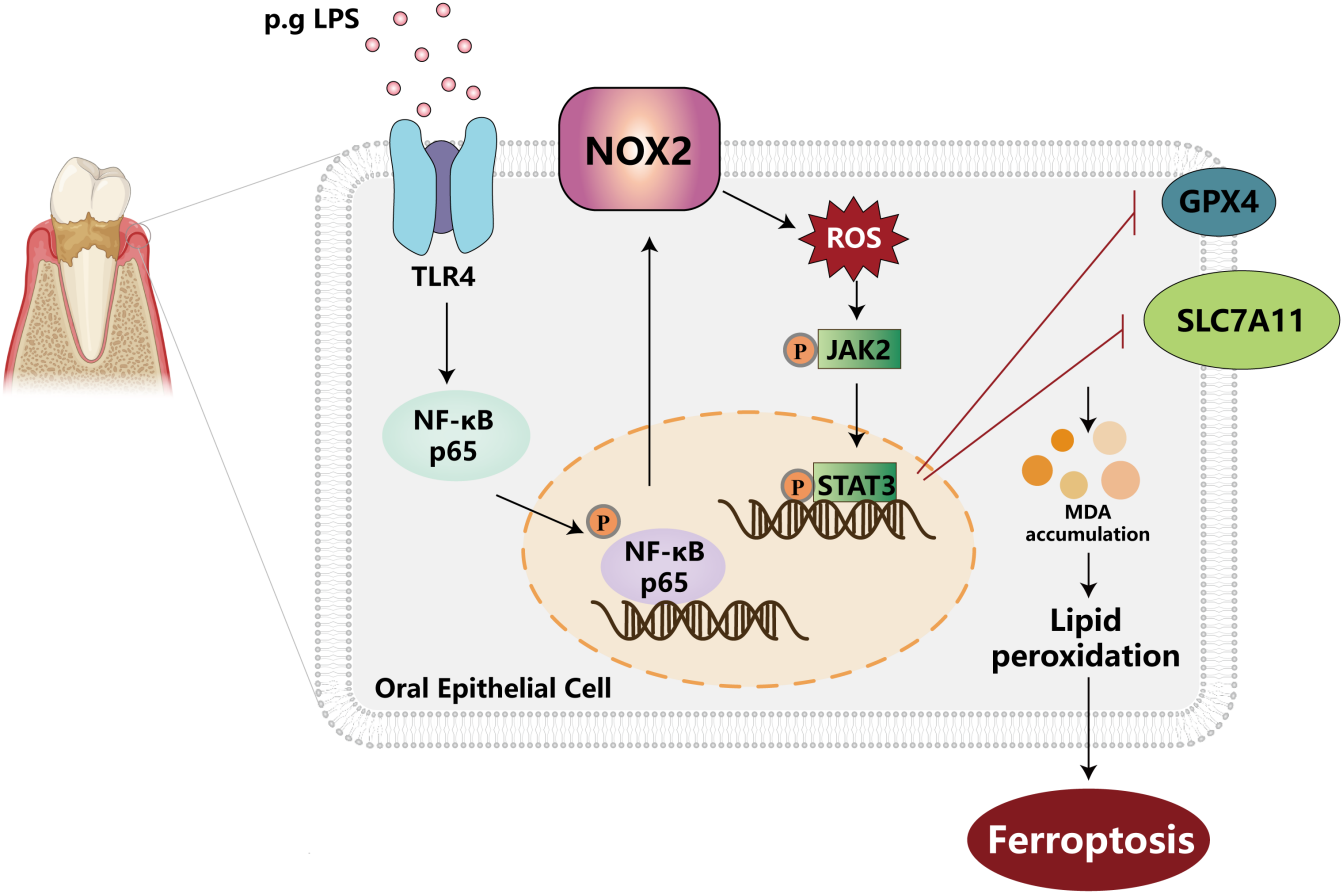
Schematic diagram illustrating the mechanism of NOX2-mediated ferroptosis in gingival epithelial cells.

Periodontitis is a chronic infectious disease caused by the microorganisms in dental plaque and represents the primary cause of tooth loss in adults (1, 50). While inflammation and oxidative stress are widely recognized as its core mechanisms, the specific forms of programmed cell death and their regulatory networks in periodontitis remain incompletely elucidated (51, 52). Ferroptosis is a novel form of regulated cell death triggered by iron-dependent lipid peroxidation, differing from other cell death types in its morphology, genetics, and biochemistry (9). Through bioinformatic analysis, we identified the *cybb* encoding NOX2 as a ferroptosis-related gene closely associated with periodontitis. *In vivo* and *in vitro* experiments further confirmed that NOX2 was highly expressed in gingival tissues of periodontitis patients as well as experimental mice and in Pg-LPS-stimulated human gingival epithelial cell CA9-22, respectively. Inhibition or knockdown of NOX2 attenuated lipid peroxidation and ferroptosis in both periodontitis mice and cellular models, thereby ameliorating gingival tissue damage and alveolar bone resorption in mice. Mechanistically, our study revealed that Pg-LPS upregulated NOX2 expression via the TLR4/NF-κB signaling pathway and NOX2-derived ROS subsequently activated the JAK2-STAT3 signaling axis to regulate ferroptosis. These findings provide novel insights into the pathogenesis of periodontitis and highlight NOX2 as a potential therapeutic target (**Figure 6**).

NOX2 is one of the major subtypes of NADPH oxidase and its core function is ROS generation that contributed to immune defense (53). Previous studies have demonstrated that NOX2 is highly expressed in periodontitis, but whether and how it regulates specific cell death patterns was unclear (28, 29, 54). Additionally, previous studies on NOX2 focused primarily on phagocytic cells such as macrophages and neutrophils. However, accumulating evidence suggested that NOX2 was also expressed in other cell types, including endothelial and epithelial cells (55–57). In our study, immunohistochemical analysis demonstrated that NOX2 was predominantly and highly expressed in the epithelial region of gingival tissue in mice with experimental periodontitis. Therefore, we established a cellular model of periodontitis by stimulating human gingival epithelial cells with Pg-LPS, and observed a significant upregulation of NOX2. The gingival epithelium serves as the primary physical and immune barrier of the oral mucosa, separating dental plaque in the oral cavity from the underlying connective tissues. The death of gingival epithelial cells is a key driver in the onset and progression of periodontitis. Microbial invasion of the gingival epithelial tissue leads to cell death and barrier disruption, thereby activating immune cells and exacerbating the inflammatory response. This process ultimately results in the destruction of the periodontal ligament and alveolar bone resorption (58, 59). In this model, Pg-LPS had been observed to induce ferroptosis. In conjunction with this, NOX2 gene silencing reduced ROS generation, suppressed lipid peroxidation, upregulated ferroptosis-related protein expression, and consequently inhibited ferroptosis in gingival epithelial cells. Furthermore, NOX2 activation also contributed to tissue inflammation and destruction. Elevated NOX2 expression has been documented in various diseases such as Alzheimer’s, Parkinson’s and atherosclerosis (60, 61). Conversely, inhibition or knockout of NOX2 could ameliorate inflammatory damage. For instance, Xu et al. found that the NOX2 inhibitor GSK2795039 reduced ROS levels in periodontal tissues, significantly reduced periodontal and intestinal inflammation, and mitigated disruption of the microbiota in both sites (62). Here, to suppress NOX2 expression in the gingival tissues of periodontitis mice, we administered the specific NOX2 inhibitor gp91 ds-tat via gingival injection. As expected, this treatment reduced lipid peroxide levels and increased ferroptosis-related protein expression, thereby suppressing ferroptosis. Collectively, this intervention ameliorated gingival inflammation and tissue damage, while also reducing alveolar bone resorption.

Mechanistically, we thoroughly investigated the regulation and effects of NOX2. We discovered that Pg-LPS promoted NOX2 expression via TLR4/NF-κB signaling pathway. *P.* gingivalis is a major periodontal pathogen and its LPS is a key virulence factor that has been widely used in periodontitis research (63–65). TLR4 is a member of the Toll-like receptor family, capable of specifically recognizing LPS from Gram-negative bacteria (66). Upon binding, LPS is presented to soluble CD14, cleaved into monomeric molecules, and then transferred to TLR4 to form a complex, activating multiple signaling pathways involved in disease progression (67). Among these, NF-κB is a key downstream pathway regulated by TLR4 and participates in modulating inflammatory responses, apoptosis, and oxidative stress processes (68). Josef et al. found that the expression of gp91phox (the primary subunit of NOX2) in mouse monocytes and microglia depended on the presence of the NF-κB p65 subunit (47). Therefore, we hypothesized that the expression of NOX2 in human gingival epithelial cells might also be regulated by p65. As anticipated, stimulation of CA9–22 with Pg-LPS upregulated TLR4 expression and promoted the phosphorylation of NF-κB p65. Pretreatment with PDTC (NF-κB inhibitor) significantly suppressed p-p65 and consequently reversed NOX2 upregulation.

ROS have been implicated in periodontitis as have NOX isoforms as the primary ROS source in periodontal tissues (29). Although NOX family proteins share similar structures and enzymatic functions, the type of NOX activation varies across different diseases. For instance, NOX1 mediates oxidative stress to alter glucose metabolism in cardiac cells (69). NOX4 promotes the proliferation, metastasis, invasion and drug resistance of numerous malignant tumors including colorectal cancer, gastric cancer, breast cancer and hepatocellular carcinoma, and is associated with poor a prognosis (70–72). NOX5 activation promotes cerebral edema, infarction and ultimately worsens neurological function following ischemia (73). In our study, we found that Pg-LPS-stimulated CA9–22 cells did not express NOX4 and no significant changes were observed in NOX1/3/5 or DUOX1/2 expression. In contrast, NOX2 was markedly upregulated, suggesting that ROS may derive from NOX2 in the cell model. ROS comprise a diverse family of molecules that acquire an extra electron in oxygen to generate superoxide anion (O_2_^−^) that is subsequently converted by superoxide dismutase into H_2_O_2_ (74). While moderate ROS levels are essential for maintaining physiological functions, their excessive accumulation leads to oxidative damage and accumulating evidence also links ROS to ferroptosis. On one hand, intracellular Fe^2+^ can generate highly reactive hydroxyl radicals via the Fenton reaction, leading to lipid peroxidation and ferroptosis. On the other hand, ROS can modulate ferroptosis through multiple signaling pathways. For example, Zhang et al. reported that a ROS-mediated oxidation-O-GlcNAcylation cascade regulates ferroptosis in hepatocellular carcinoma (75), while Liu et al. demonstrated that TIGAR induces ferroptosis resistance in colorectal cancer cells via the ROS/AMPK/SCD1 pathway (76).

To further investigate the mechanism by which NOX2 regulates ferroptosis, we performed gene set enrichment analysis (GSEA) on gingival tissue samples from periodontitis patients (GSE16134). The results demonstrated significant enrichment of genes of the JAK–STAT pathway in samples with high NOX2 expression. The JAK/STAT signaling pathway is one of the most ubiquitous intracellular signal transduction systems, composed of tyrosine kinase-associated receptors, Janus kinases (JAK), and signal transducers and activators of transcription (STAT) (77). It regulates diverse cellular processes including proliferation, differentiation, apoptosis, and immune responses (78). Upon activation, JAKs phosphorylate specific tyrosine residues on STAT proteins, leading to STAT dimerization, nuclear translocation, binding to specific DNA sequences, and regulation of downstream target gene expression (78). Notably, NOX2 shows a particularly close association with JAK2 and STAT3 within this pathway. For instance, hepatocyte growth factor promotes a proangiogenic phenotype and mobilizes endothelial progenitor cells via NOX2 activation, whereas JAK2 inhibition suppresses tube formation in human umbilical vein endothelial cells (79). Additionally, NOX2 disrupts VEGF-A-induced angiogenesis in placental tissue through a mitochondrial ROS–STAT3 mechanism (80). Moreover, NOX2 can interact directly with STAT3 in human trophoblast cells to modulate ferroptosis (35). Additional studies have also indicated that STAT3 plays a context-dependent role in ferroptosis. Dong et al. reported that JAK/STAT3 pathway inhibition alleviates cisplatin-induced ferroptosis and protects against renal injury (81). In contrast, Li et al. showed that FANCD2 activates the JAK2/STAT3 axis to suppress ferroptosis in human osteosarcoma cells (82). Integrating these findings with our bioinformatic analyses, we hypothesized that NOX2 modulates ferroptosis in periodontitis via JAK2–STAT3 signaling. To test this hypothesis, we examined JAK2 and STAT3 expression in Pg-LPS-induced cells and employed H_2_O_2_ to simulate ROS effects *in vitro*. Consistent with our hypothesis, NOX2 knockdown under inflammatory conditions markedly reduced the phosphorylation levels of both JAK2 and STAT3. Additionally, H_2_O_2_ can activate JAK2 and STAT3 while downregulating the anti-ferroptotic proteins GPX4 and SLC7A11. Consequently, JAK2 inhibition was also found to suppress H_2_O_2_-induced ferroptosis, suggesting that GPX4 and SLC7A11 may be transcriptional targets of STAT3. This inference aligns with the findings of Ouyang et al. in gastric cancer research that demonstrated that STAT3 can directly bind to GPX4 and SLC7A11 gene promoter regions (83). Collectively, these results indicated that in periodontitis, NOX2-derived ROS may promote ferroptosis via the JAK2-STAT3 signaling pathway.

Although this study provides compelling evidence, several limitations should be acknowledged, which also point to directions for future research. First, the mechanistic exploration was primarily conducted in the immortalized gingival epithelial cell line CA9-22, and the applicability of these findings to primary gingival epithelial cells and other periodontal-related cell types requires further validation. In future studies, we will isolate and utilize primary human gingival epithelial cells to verify the relevant results. Second, the cellular experiments employed Pg-LPS as a single inflammatory stimulus, whereas periodontitis is essentially a polymicrobial infectious disease. Therefore, whether the conclusions apply to other periodontal pathogens or microbial communities remains to be further confirmed. In subsequent work, we will employ LPS from other key periodontal pathogens such as *Prevotella intermedia* and *Fusobacterium nucleatum* to conduct parallel comparative experiments to systematically evaluate the broad relevance of this signaling pathway. Furthermore, at the animal experimental level, although the NOX2 inhibitor gp91 ds-tat demonstrated ameliorative effects on periodontitis, the activation status of JAK2/STAT3 was not simultaneously examined in this treatment group, nor were JAK2 inhibitors or ferroptosis inhibitors applied for *in vivo* intervention. Thus, whether NOX2 precisely regulated ferroptosis through the same pathway *in vivo* still requires more direct supporting evidence. In the next step, we will combine an epithelial cell-specific conditional NOX2 knockout mouse model with downstream pathway inhibitors to provide more robust genetic evidence for this mechanism *in vivo*.

In summary, our study integrated bioinformatic analysis and experimental validation to identify NOX2 as a pivotal ferroptosis-related gene in periodontitis. We confirmed the upregulation of NOX2 in human, animal and cellular models and that it promotes periodontitis via ferroptosis. Mechanistically, NOX2 drives ferroptosis in gingival epithelial cells via the ROS/JAK2-STAT3 signaling and thereby exacerbates periodontitis severity. Our findings highlight that targeting NOX2 inhibition holds promise as a novel therapeutic strategy for periodontitis.

## Data Availability

The datasets presented in this study can be found in online repositories. The names of the repository/repositories and accession number(s) can be found in the article/**Supplementary Material**.
