# Supplementary material for: NOX2 exacerbates periodontitis via JAK2-STAT3-mediated ferroptosis of gingival epithelial cells
Source: Front Immunol. 2026 Feb 26;17:1744612. doi: 10.3389/fimmu.2026.1744612 (PMC12979118; doi:10.3389/fimmu.2026.1744612)
Supplement: Supplementary file 1 [file DataSheet1.docx]

**Supplemental Materials**


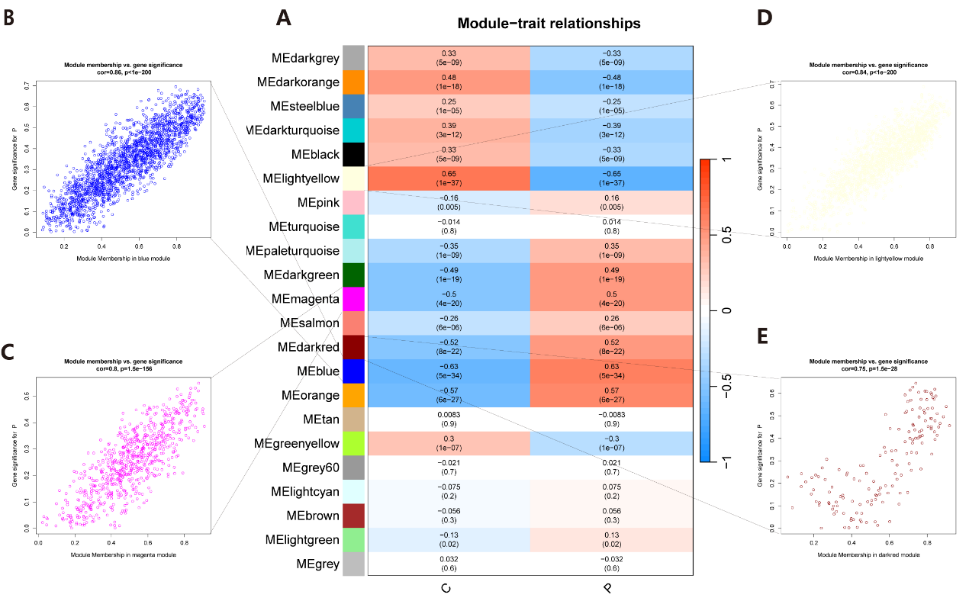


**Fig.S1 Genes related to periodontitis identified by WGCNA screening of the GEO dataset GSE16134. (**A) Association of gene modules with clinical traits. (B-E) Correlation between module membership and gene significance.


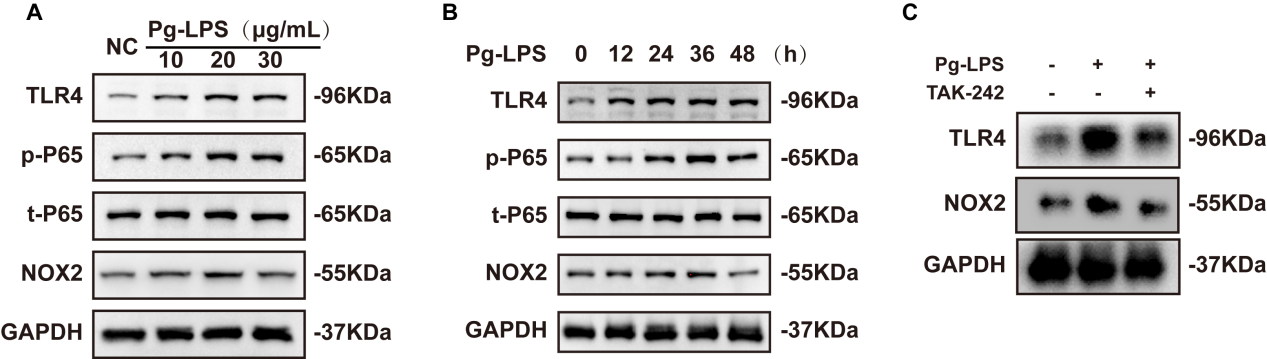


**Fig.S2 Pg-LPS upregulates NOX2 expression via the TLR4/ NF-κB signaling pathway.** (A) Western blot analysis of TLR4, p-p65, t-p65, and NOX2 protein levels in CA9-22 cells treated with Pg-LPS (10, 20, 30 μg/mL) for 24 h. (B) Western blot analysis of protein levels in CA9-22 cells treated with Pg-LPS at 0, 12, 24, 36 and 48 h. (C) CA9-22 cells were pretreated with TAK-242 (1 μM) for 2 h before the addition of 20μg/mL Pg-LPS for 24 h; NOX2 and TLR4 protein levels were measured by Western blotting.


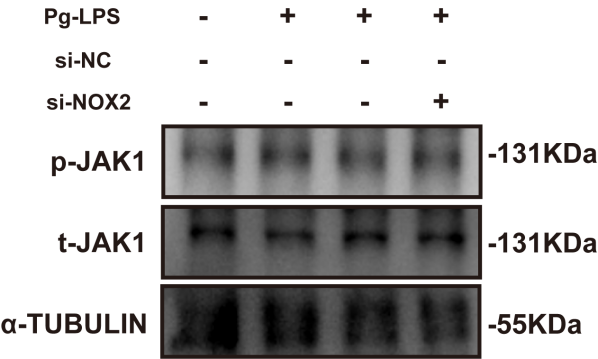


**Fig.S3 Phosphorylation levels of JAK1 in CA9-22 cells after NOX2 gene silencing.**


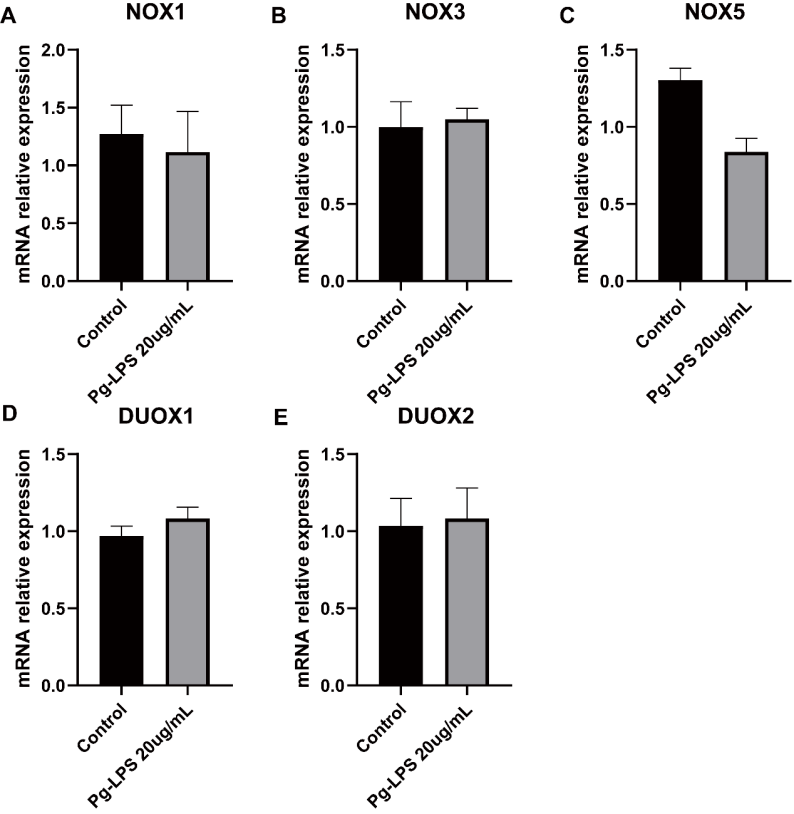


**Fig.S4** (A-E) RT-qPCR measurements of mRNA levels for NOX1, NOX3, NOX5, DUOX1 and DUOX2 in Pg-LPS stimulated CA9-22 cells.


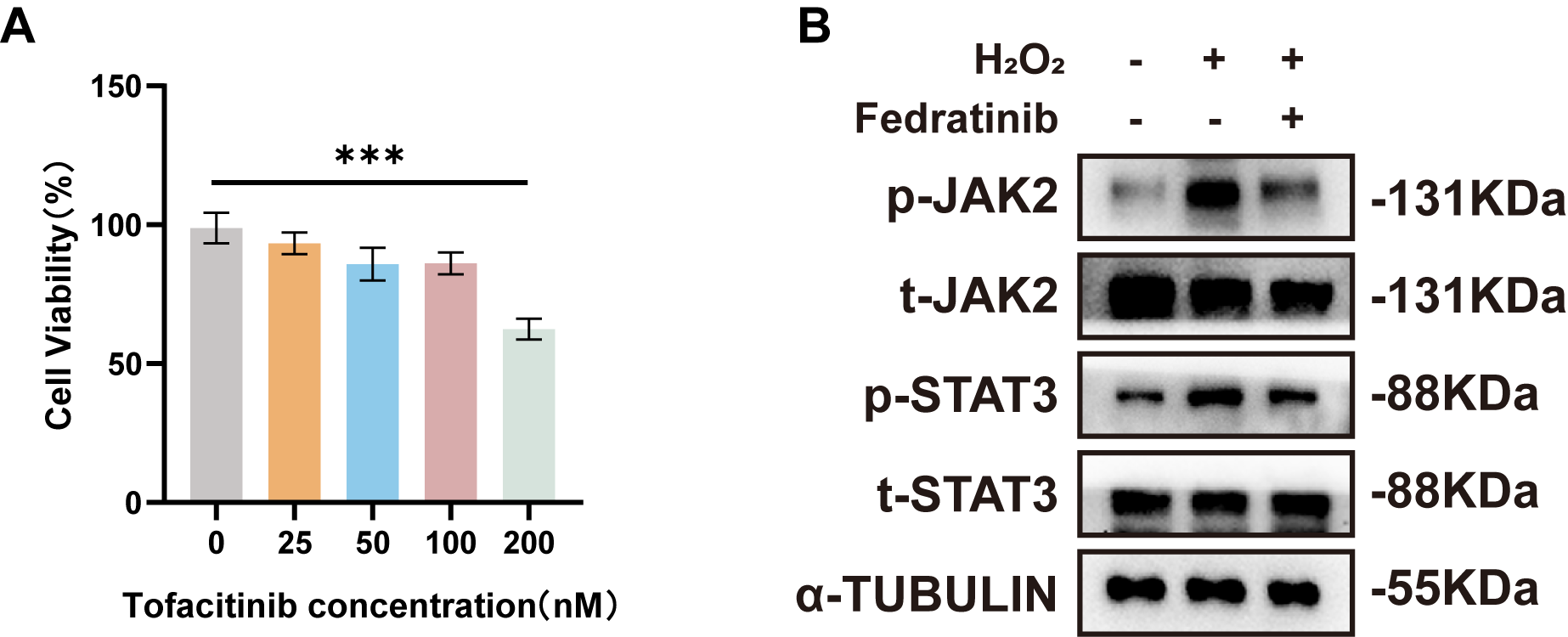


Fig.S5 (A) Cell viability determined using the CCK8 assay. (B) CA9-22 cells were pretreated with Fedratinib (3 μM) for 1 h before the treatment of 400 μM H_2_O_2_ for 6 h; the levels of JAK2 and STAT3 were measured by Western blotting.


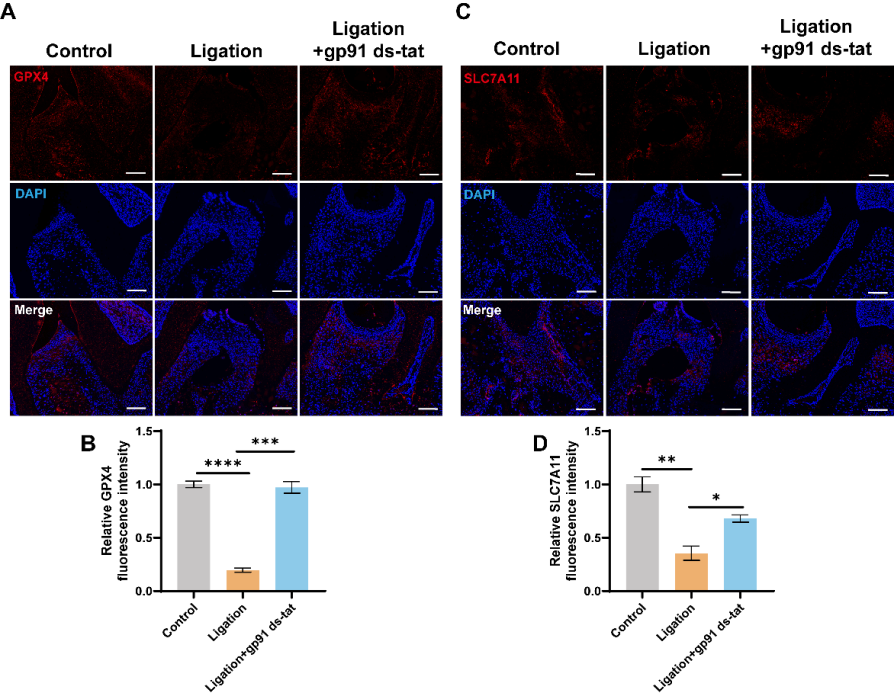


**Fig.S6** (A-B) Immunofluorescence analysis of GPX4 (Scale bars: 100 μm). (C-D) Immunofluorescence analysis of SLC7A11 (Scale bars: 100 μm).
